# Effectiveness of Pulpotomy in the Management of Symptomatic Irreversible Pulpitis in Mature Permanent Teeth: An Umbrella Review

**DOI:** 10.7759/cureus.104814

**Published:** 2026-03-07

**Authors:** Maria Mtalsi, Mouna El Asmar, Manal Abouelala, Samira El Arabi

**Affiliations:** 1 Department of Pediatric Dentistry, Faculty of Dental Medicine, Hassan II University, Casablanca, MAR; 2 General Dentistry, Private Practice, Casablanca, MAR

**Keywords:** child, irreversible pulpitis, mature permanent teeth, pulpotomy, systematic review, vital pulp therapy

## Abstract

The objective of this umbrella review is to analyze the conclusions drawn from systematic reviews and meta-analyses on pulpotomy effectiveness in the treatment of irreversible pulpitis in mature permanent teeth, focusing on the impact of pulp capping materials and factors influencing treatment outcomes.

A search of electronic databases (PubMed, Science Direct, Google Scholar, CINAHL, Cochrane Library) was conducted up to December 2022 using the keywords: Pulpotomy, Child, Permanent mature teeth, Irreversible pulpitis. The methodological quality of systematic reviews was assessed using the AMSTAR (A Measurement Tool to Assess Systematic Reviews) 2 tool.

Of the 332 articles selected, 11 were chosen for data extraction, focusing on pulpotomy success in treating irreversible pulpitis in mature permanent teeth, and evaluating the influence of materials and techniques on outcomes. Applying AMSTAR 2 criteria, only one systematic review was rated “moderate” quality; most were rated as “low” to “very low” quality.

This umbrella review reported a very high pulpotomy success rate, up to 100%, varying with materials and follow-up duration. Pulpotomy success rates range from 37% to 100%, depending on the material used, with Biodentine® (Septodont, Saint-Maur-des-Fossés, France), achieving the highest success rate.

Short-term pulpotomy success rates ranged from 80% to 95%, exceeding root canal treatment, while long-term outcomes remain comparable. Pulpotomy success depends on pre- and perioperative conditions, hemostasis, and definitive coronal restoration.

Analysis of systematic reviews indicates that pulpotomy is a promising alternative to conventional root therapy for managing irreversible pulpitis in mature permanent teeth. Available evidence remains limited, emphasizing the need for high-quality trials to confirm its effectiveness.

## Introduction and background

Irreversible pulpitis has long been defined as pulp damage that cannot heal, clinically manifested by intense and persistent spontaneous pain exacerbated by thermal pulp tests [1]. Root canal treatment has always been the preferred therapeutic option for irreversible pulpitis, even in cases where root canal treatment is complex. These therapeutic approaches are based on the traditional understanding of pulpitis pathophysiology, particularly the “pulp strangulation theory,” which suggests that the entire pulp is considered incapable of healing [2]. However, recent histopathological investigations have shown that inflammation in teeth presenting irreversible pulpitis is typically confined to the coronal pulp, and that radicular pulp tissue remains unaffected and may be preserved. These observations have demonstrated a weak correlation between the clinical diagnosis and the true histological state of the pulp in permanent teeth with closed apex. Thus, a tooth that clinically presents with irreversible pulpitis does not necessarily present with it histologically, with the possibility of having part of the pulp healthy or presenting with reversible inflammation [3,4]. 

The shift towards minimally invasive endodontics, enhanced knowledge of pulp defense responses, the availability of bioactive medications, and accumulating clinical and radiographic evidence have led to pulpotomy being recognized as a viable alternative to conventional root canal treatment, including in mature teeth [5].

According to the American Association of Endodontists (AAE), pulpotomy is now defined as the removal of the coronal portion and preservation of the remaining root portion [1]. Its purpose is to maintain pulp vitality by selectively removing infected/inflamed tissue and preserving healthy, functional tissue. In addition to its conservative philosophy, pulpotomy has several advantages: preservation of the tooth structure and, consequently, increased tooth survival, reduction of symptoms (pain and discomfort) for the patient, simplification of clinical procedures, reduced costs for patients, and preservation of the immunological and proprioceptive functions of the remaining pulp [5,6].

The past decade has witnessed a notable growth in clinical research and review articles investigating the effectiveness of pulpotomy in managing irreversible pulpitis in mature permanent teeth. Whereas before, pulpotomy was generally used as an emergency treatment to alleviate the pain of irreversible pulpitis of mature permanent teeth. The majority of these studies have focused on the effect of different capping materials on the preservation of pulp vitality in mature permanent teeth after pulpotomy.

In this context, this umbrella review aims to critically analyze the systematic reviews and meta-analyses published on pulpotomy of mature permanent teeth with irreversible pulpitis in order to clarify the clinical and radiographic efficacy of this approach, evaluate the influence of bioactive materials, and identify other factors that may influence the success or failure of this therapy.

## Review

Methods

Protocol and Registration

This umbrella review was conducted in accordance with the PRISMA (Preferred Reporting Items for Systematic Reviews and Meta-Analyses) guidelines for systematic reviews and meta-analyses [7]. The protocol was registered in the PROSPERO database under reference (CRD42024500772) to minimize the risk of unplanned duplication of the research on this topic, in accordance with the PRISMA 2009 statement. The research question was formulated as follows: How effective is pulpotomy in the treatment of irreversible pulpitis in mature permanent teeth?

Inclusion Criteria

Systematic reviews, with or without meta-analyses, that met PRISMA criteria and were published in English were included. These reviews had to evaluate the effectiveness or success of pulpotomy in the treatment of mature permanent teeth with irreversible pulpitis in children and/or adults. No restrictions were applied to the year of publication.

Exclusion Criteria

Publications whose full text was not accessible and those that did not meet the predefined inclusion criteria were excluded.

Research Strategy

An electronic data search was conducted across five electronic databases: PubMed, Google Scholar, Science Direct, CINAHL, and the Cochrane Database of Systematic Reviews. The Population, Intervention, Comparison, and Outcome (PICO) framework was adopted to define the eligibility criteria of the included studies. The population consisted of children or the general population presenting mature permanent teeth affected by caries and irreversible pulpitis. The intervention of interest was pulpotomy, while the comparison was pulpectomy. The primary outcome was healing, defined by asymptomatic tooth and the absence of pulp and periodontal complications.

Keywords and headings were merged according to each database's thesaurus, and split headings were applied. The search was based on references from systematic reviews and meta-analyses. The Boolean search strategy was structured around four concepts: Permanent Dentition AND/OR Child AND Irreversible Pulpitis AND Pulpotomy. Multiple combinations of these blocks were tested sequentially, and the final combination was selected to minimize irrelevant results while generating a manageable number of records for screening, ensuring transparency and reproducibility. The electronic search was conducted over a period of three months, from October 2022 to December 2022, and was limited to English-language studies.

Data Collection

Two reviewers independently screened all titles and abstracts. The same researchers then reviewed the full text of studies deemed relevant or potentially relevant based on the review of titles and abstracts. Any disagreements were resolved through discussion with a third reviewer.

Data Extraction

Data from all included studies were extracted and assessed using data extraction forms designed for this purpose. For each review, the following data were systematically recorded: authors, year of publication, number of studies included, study design, population, exposure, comparison, variables studied, methods of analysis and quality assessment, and results (main results and main conclusions).

Risk of Bias Assessment

Two researchers used the AMSTAR 2 measurement tool to assess the systematic reviews in order to determine the methodological quality of the reviews included. This 16-item instrument allows for an assessment of the overall confidence in the results reported in the systematic reviews [8].

Results

Study Selection

**Figure 1:**
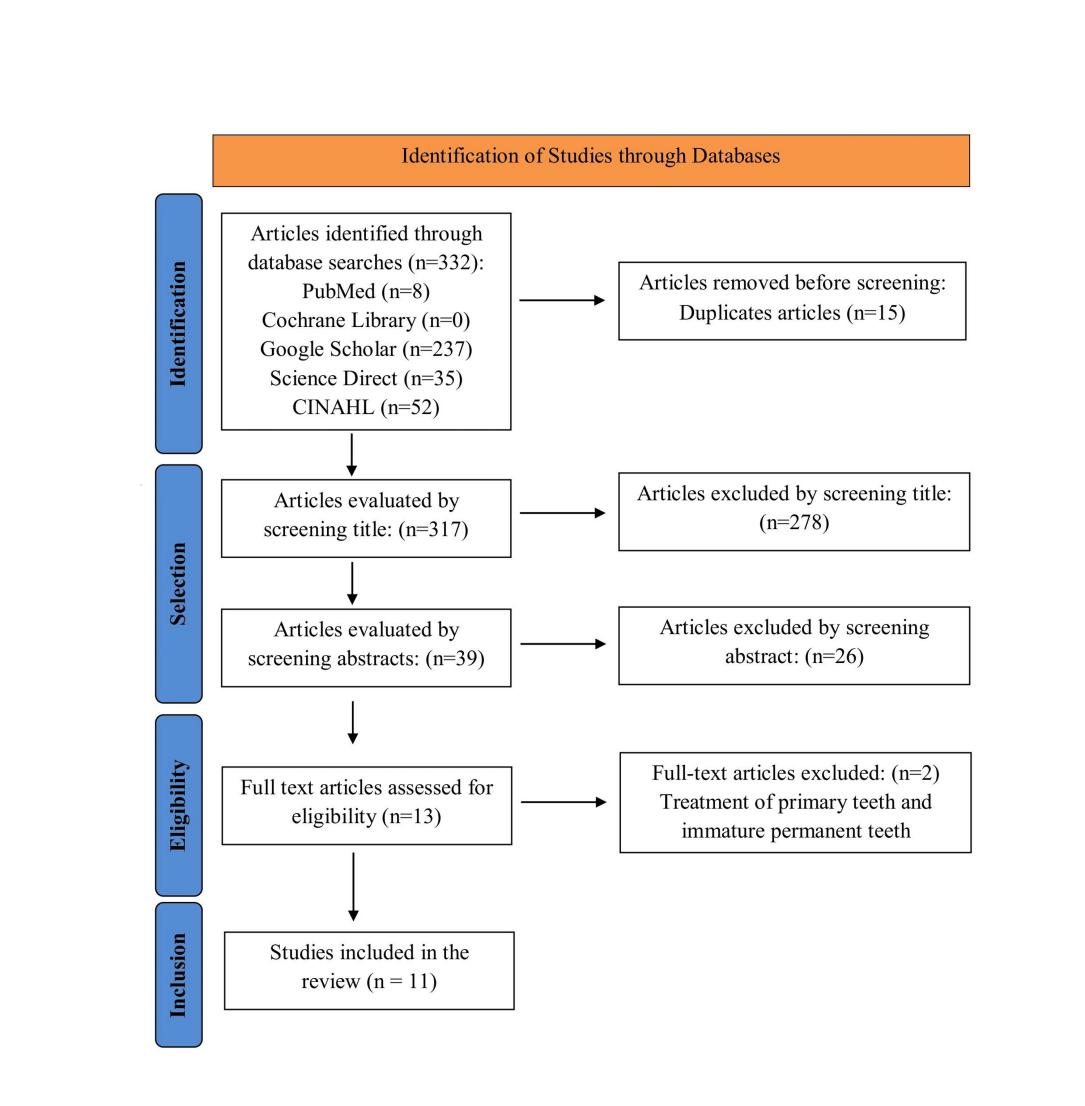
PRISMA flow diagram of identification and selection of included reviews PRISMA: Preferred Reporting Items for Systematic Reviews and Meta-Analyses

The initial literature search identified a total of 332 articles. After removing 15 duplicates, 304 articles were excluded based on a review of the titles and abstracts. The remaining 13 articles were analyzed in full text, and two were excluded because they dealt with primary teeth or immature permanent teeth. Finally, 11 systematic reviews were deemed eligible and included in the analysis (Figure 1).

Study Characteristics

A total of 11 systematic reviews were included in this umbrella review (Table 1). The systematic reviews covered the period between 2018 and 2022 and evaluated the effectiveness of pulpotomy in the treatment of irreversible pulpitis in mature permanent teeth by comparing different capping materials and reporting clinical and radiographic outcomes.

**Table 1:** Characteristics of the systematic reviews included SR: systematic review; MA: meta-analysis; RCT: root canal treatment; GRADE: Grading of Recommendations Assessment, Development and Evaluation

Authors/year	Types and numbers of studies included	Qualitative assessment tools	Analysis method	Variables studied
Skitioui et al. (2022) [9]	4 randomized studies	-	SR	- Comparison between RCT and pulpotomy. - Comparison between different pulpotomy materials.
Zanini et al. (2019) [10]	- 15 ex vivo histological studies - 15 case reports or case series - 17 cohort studies - 10 clinical trials or randomized clinical trials	The GRADE approach	SR	Procedures and materials applied for pulp capping and crown restorations following complete pulpotomy on mature permanent teeth with irreversible pulpitis.
Tomson et al. (2022) [11]	5 randomized studies	- The GRADE approach - Cochrane Risk of Bias Tool	SR and MA	Effectiveness of pulpotomy versus RCT in managing post-operative pain in permanent teeth with irreversible pulpitis.
Vibha et al. (2022) [12]	13 (in vivo clinical trials, retrospective studies, randomized controlled trials)	Cochrane Risk of Bias Tool	SR and MA	Effectiveness of pulpotomy compared to RCT in relieving pain from symptomatic irreversible pulpitis and its treatment outcome on mature permanent posterior teeth.
Hakami et al. (2020) [13]	8 (prospective studies)	Cochrane Risk of Bias Tool	SR	Evaluation of the pulpotomy procedure in the treatment of irreversible pulpitis of mature permanent teeth.
Taneja and Singh (2019) [14]	5 (randomized controlled trials)	Cochrane Risk of Bias Tool	SR and MA	Effectiveness of MTA and calcium hydroxide as pulpotomy agents on permanent teeth.
Santos et al. (2021) [15]	12 (randomized controlled trials, prospective cohort studies)	- ROBINS-I tools and Newcastle-Ottawa scale - Cochrane Collaboration’s tool	SR	Outcomes of vital pulp therapy on mature permanent posterior teeth with symptomatic irreversible pulpitis.
Ather et al. (2022) [16]	11 (prospective and retrospective studies, randomized controlled trials)	- Cochrane Risk of Bias Tool 2 (RoB 2) - Modified Down and Black checklist	SR and MA	Evaluation of the success rate of pulpotomy in carious teeth diagnosed with irreversible pulpitis, and analysis of the influence of predictive factors such as symptoms, root apex development and type of pulp capping material on the treatment outcomes.
Zafar et al. (2020) [17]	6 (randomized controlled trials, single-group intervention studies, and retrospective studies)	Cochrane Risk of Bias Tool	SR	Evaluation of the success of pulpotomy on mature permanent teeth with irreversible pulpitis.
Li et al. (2019) [18]	21 (randomized controlled trials)	- Cochrane Manual for Systematic Reviews of Interventions 5.1.0 - GRADE	SR and MA	Effectiveness and cost-efficiency of pulpotomy in permanent teeth with pulp exposure due to extensive caries.
Cushley et al. (2019) [19]	8 (prospective and retrospective studies, randomized controlled trials)	- Cochrane Handbook for Systematic Reviews of Interventions - Modified Downs and Black quality assessment checklist	SR	Comparison between pulpotomy and RCT.

Risk of Bias Assessment

Good inter-examiner reliability was recorded despite a risk of selection bias (kappa score = 1.000). Regarding the methodological quality of the systematic reviews, six studies were considered to be of extremely low quality [9-14], four were of poor quality [15-18], and only one was of moderate quality [19]. The results reflect limited confidence in the available conclusions (Table 2).

**Table 2:** Qualitative assessment of the risk of bias in the included systematic reviews based on the AMSTAR 2 criteria AMSTAR: A Measurement Tool to Assess Systematic Reviews

Authors/year	Quality of review
Skitioui et al. (2022) [9]	Critically low
Zanini et al. (2019) [10]	Critically low
Tomson et al. (2022) [11]	Critically low
Vibha et al. (2022) [12]	Critically low
Hakami et al. (2020) [13]	Critically low
Taneja and Singh (2019) [14]	Critically low
Santos et al. (2021) [15]	Low
Ather et al. (2022) [16]	Low
Zafar et al. (2020) [17]	Low
Li et al. (2019) [18]	Low
Cushley et al. (2019) [19]	Moderate

Summary of Results

The studies included in this umbrella review showed a very high success rate for pulpotomy, reaching up to 100%. However, this success rate varies considerably depending on the materials used and the duration of follow-up. The materials used play an important role in the success of pulpotomy on mature permanent teeth with irreversible pulpitis. Pulp therapy with calcium hydroxide (CH) has the lowest success rate, which can be as low as 37%. Mineral Trioxide Aggregate (MTA) has a high clinical success rate of between 82% and 100%, with a lower radiographic success rate of between 53.3% and 95%. Calcium-enriched cement (CEM) has shown a clinical success rate of between 86% and 100%, while its radiographic success rate is between 86% and 98%. Biodentine® (Septodont, Saint-Maur-des-Fossés, France), meanwhile, has a high success rate of between 80% and 100%.

The articles included in this umbrella review discussed the factors influencing the success of pulpotomy in mature permanent teeth with irreversible pulpitis, namely perioperative conditions, hemostasis, pulp healing potential, the presence or absence of periapical involvement, and patient age.

The success of pulpotomy was observed in the short term (≤12 months) with healing rates of 80% to 95% [9,12], which was higher than conventional root canal treatment, while in the long term, there was no difference between the two therapies. However, the authors suggested that pulpotomy yielded better results than conventional root canal treatment in the management of postoperative pain.

Table 3 summarizes the main conclusions drawn from the 11 systematic reviews.

**Table 3:** Main conclusions drawn from the included reviews RCT: root canal treatment; CEM: calcium-enriched mixture cement; MTA: mineral trioxide aggregate; CH: calcium hydroxide; PRF: platelet-rich fibrin; ZOE: zinc oxide eugenol; IRM: intermediate restorative material (reinforced zinc oxide eugenol)

Author/year	Population	Intervention	Comparison	Clinical sucess (%)	Radiographic sucess (%)		Main conclusion
Skitioui et al. (2022) [9]	1,281 teeth affected by irreversible pulpitis (age range between 9 and 65 years old)	Pulpotomy with CEM, MTA, CH, PRF	Root canal treatment	CEM: 97-98.19%	CEM: 86.1- 92.2%		Pulpotomy can be considered as a valid optional treatment for irreversible pulpitis in mature permanent teeth, and its success depends on the healing potential of the remaining pulp and on the biocompatibility and bioactivity of pulp capping materials.
				CH: 81.2%	CH: 46.1%		
				MTA: 83.3-98%	MTA: 53.3- 95%		
Zanini et al. (2019) [10]	Patients with irreversible pulpitis in mature permanent teeth	Pulpotomy with CH, CH covered with ZOE or IRM, MTA, CEM, ZOE, Biodentine, PRF	undescribed	CH and CH covered with ZOE or MRI: 37 - 100%	-		There is no single recommended procedure for full pulpotomy in vital permanent teeth due to varying pulpal diagnoses from healthy pulp to irreversible pulpitis. Essential steps, including preventing contamination, controlling infection, and achieving a complete seal, should be followed.
				Biodentine: 80-100%			
				MTA: 44-100%			
				CEM: 78-100%			
				ZOE: 42-100%			
				PRF: 100%			
Tomson et al. (2022) [11]	2178 patients with mature molars affected by irreversible pulpitis (aged between 9 and 65 years)	Pulpotomy with CEM	Root canal treatment	At 12 months			There is no difference in patient-reported pain between RCT and pulpotomy at Day 7 postoperatively, and a single clinical trial suggests similar long term clinical success for both procedures.
				Pulpotomy: 97.6%	Pulpotomy: 92.8%		
				RCT: 98.3%	RCT: 81.2%		
				At 2 years			
				Pulpotomy: 98.2%	Pulpotomy: 86.1%		
				RCT: 79.5%	RCT: 79.5%		
				At 5 years			
				Pulpotomy: 78.1%			
				RCT: 75.3%			
Vibha et al. (2022) [12]	1234 patients with teeth affected by irreversible pulpitis (age range between 6 and 79 years old)	Pulpotomy with CEM CH BIODENTINE ZOE PRF MTA Dexamethasone	Root canal treatment	Full pulpotomy MTA: 80-85%	-		Pulpotomy can be considered an alternative option for mature permanent teeth with irreversible pulpitis, allowing preservation of tooth vitality and providing benefits such as reduced treatment time, decreased postoperative pain, and simplified overall arsenal.
				Partial pulpotomy MTA: 85-90%			
				Partial pulpotomy BIODENTINE: 90%			
				Partial pulpotomy CH: 43%			
Hakami et al. (2020) [13]	1547 patients with mature permanent molars with irreversible pulpitis	Pulpotomy with CEM MTA CH	undescribed	-	-		Pulpotomy is a promising strategy for managing irreversible pulpitis across different age groups, especially for molar teeth.
Taneja and Singh (2019) [14]	244 permanent molars with irreversible pulpitis (aged 6 to 61 years)	Pulpotomy with CH	Pulpotomy with MTA	At 6 months and 12 months, the success rate of pulpotomy with MTA is significantly higher than that of pulpotomy with CH.			MTA is the superior material for pulpotomy in permanent teeth, while calcium hydroxide shows a significantly higher failure rate.
Santos et al. (2021) [15]	Mature permanent posterior teeth diagnosed with irreversible symptomatic pulpitis (age range 8 to 65 years)	Total and partial pulpotomy with CEM MTA BIODENTINE MTA CH PRF	Root canal treatment	At 1 year			Full and partial pulpotomy using hydraulic calcium silicate cements demonstrates favorable outcomes in mature permanent posterior teeth with symptomatic irreversible pulpitis.
				Full pulpotomy using MTA, CEM, or Biodentine: 92-100%		Full pulpotomy using MTA, CEM, or Biodentine: 92-98%	
				At 2 years			
				Full pulpotomy using CEM: 98%		Full pulpotomy using CEM: 86%	
				At 3-5 years			
				Full pulpotomy using MTA or CEM: 98%		Full pulpotomy using CEM or MTA: 78-85%, respectively.	
Ather et al. (2022) [16]	Patients with permanent molars affected by irreversible pulpitis	Pulpotomy with MTA BIODENTINE CH CEM	Undescribed	Pulpotomy on permanent teeth with irreversible pulpitis: 86%.			This study demonstrated that pulpotomy is an effective procedure with a high success rate for treating teeth with irreversible pulpitis.
				Teeth with open: 96%. Teeth with closed apices: 83%			
				Biodentine demonstrated a significantly higher success rate than MTA, CEM, and CH.			
Zafar et al. (2020) [17]	691 teeth affected by irreversible pulpitis (age range 10-70 years).	Pulpotomy	Root canal treatment	The overall clinical and radiographic success rate of the studies reviewed ranged from 68% to 100%.			There is no reliable tool to measure the extent of pulp inflammation, but hemostasis can indicate inflammation limited to the coronal pulp. Pulpotomy is effective in treating irreversible pulpitis and healing periapical rarefaction, with long-term follow-up recommended to monitor for complications.
Li et al. (2019) [18]	Pulp exposure due to caries on permanent teeth	Pulpotomy with CH, MTA, PRF CEM, BIODENTINE, Triple antibiotic paste	- Root canal treatment. - Direct pulp capping	Pulp exposure due to caries on permanent teeth	At 24 months: MTA had a higher radiographic success rate than CEM on mature permanent teeth		MTA appears to be the best material for pulpotomy in carious permanent teeth with pulp exposure, while pulpotomy using CEM outweighs root canal treatment. Pulpotomy using CH has a higher radiographic success rate than direct pulp capping.
					At 60 months: Pulpotomy using CH had a higher radiographic success rate than direct pulp capping.		
Cushley et al. (2019) [19]	1,447 patients (aged 9 to 65) with irreversible pulpitis, reversible pulpitis, and periapical periodontitis	Pulpotomy with MTA CEM BIODENTINE	Root canal treatment	At 12 months			Evidence indicates a high success rate for pulpotomy in teeth with symptomatic irreversible pulpitis, however, the results are based on heterogeneous studies with a high risk of bias. Well-designed, adequately powered randomized controlled trials are needed for the evidence to change clinical practice.
				97.4%	95.43%		
				At 36 months			
				93.97%	88.39%		

Discussion

This umbrella review provides a qualitative synthesis of data from systematic reviews on the effectiveness of pulpotomy in the management of mature permanent teeth affected by irreversible pulpitis. The protocol followed universally accepted guidelines. The search followed PRISMA guidelines and was limited to contemporary publications from 2018 to 2022. Strict inclusion criteria limited this review to mature permanent teeth diagnosed with irreversible pulpitis treated by pulpotomy.

Initially, we wanted to verify the effectiveness of this therapy in children. However, using “And” ‘Child’ as a search mesh on PubMed yielded zero results, which led us to use “and/or.” The selected articles cover a very wide age range, including children, adolescents, and adults. As a result, we were unable to draw conclusions about the benefits of pulpotomy on mature permanent teeth with irreversible pulpitis in children, but rather about the benefits of this therapy on mature permanent teeth regardless of the patient's age.

The relevance of this work lies in the number of reviews dealing with the subject. Nevertheless, the methodological quality of the reviews selected according to AMSTAR criteria varies from very low to moderate, which calls for caution in interpreting the results.

Effectiveness of Pulpotomy as a Definitive Treatment for Irreversible Pulpitis in Mature Permanent Teeth

According to this reading, the success of pulpotomy is often compared to the success of root canal treatment (RCT). The studies included in this umbrella review showed a very high success rate (often 80%-95%) in the short term, sometimes reaching 100%. However, this success rate varies considerably depending on the materials used and the duration of follow-up. In the short term (≤12 months), the clinical success rate of pulpotomy is generally between 80% and 95%, with some studies reporting rates close to 100%. At this stage, pulpotomy is at least as effective as non-surgical root canal treatment with additional advantages (quick procedure, less invasive, and often better tolerated by patients) [9,12].

In the long term (for a follow-up period of more than 36 months), the available data remain limited and heterogeneous. Cushley et al. (2019) report a clinical success rate of 93.97% at 36 months, but a decline to 71.3% at 60 months [19].

These results suggest a possible decrease in effectiveness over time, but interpretation must remain cautious due to the poor methodological quality of the available studies and the risk of significant bias.

In addition, four systematic reviews [9,11,12,19] in this umbrella review directly compared pulpotomy with conventional endodontic treatment. Overall, they conclude that the two treatment modalities are comparable in terms of efficacy. Some even point to better postoperative pain control with pulpotomy, while others highlight similar long-term results regardless of the technique used.

Thus, although root canal treatment remains the traditional standard of treatment, these four analyses suggest that pulpotomy is a viable therapeutic alternative for mature permanent teeth affected by irreversible pulpitis, provided that the selection criteria and surgical protocol are followed.

Pulp Capping Materials

Beyond comparing it with root canal treatment, several reviews have also examined the impact of different pulp capping materials on the success of pulpotomy, highlighting their crucial role in the long-term effectiveness of this treatment.

In fact, calcium hydroxide (CH), reported in six reviews [9,10,12,14,16,18], has the lowest success rates (sometimes as low as 37%). As for MTA, described in eight reviews [9,10,12-16,19], it shows high clinical results (82% - 100%), but more variable radiographic rates (53.3% to 95%). Calcium-enriched mixture cement (CEM), cited in six reviews [9,11,15,16,18,19], achieves clinical and radiographic success rates of over 85% with performance similar to MTA. Platelet-rich fibrin (PRF), mentioned in three reviews [9,12,20], shows heterogeneous results ranging from 35.5% to 100% success rates, and its use remains experimental. Finally, Biodentine, cited in five reviews [10,12,15,16,19], appears to be the material that offers the highest success rates, between 80% and 100%, with a lower risk of discoloration.

In summary, calcium silicate-based hydraulic cements (MTA, CEM, Biodentine) offer superior and more consistent results than calcium hydroxide, although it is not possible to definitively state that one material is superior to another.

Factors Contributing to the Success/Failure of a Pulpotomy

Based on a review of all the articles, there are many factors that influence the success of pulpotomy on mature permanent teeth with irreversible pulpitis.

Perioperative conditions: these include preoperative decontamination, the type of anesthetic (lidocaine or articaine with epinephrine), isolation with a rubber dam, the technique used for curettage of carious tissue, and perioperative irrigation for disinfection or hemostasis with sodium hypochlorite or chlorhexidine solutions [10,15,21,22].

Hemostasis: Regardless of the hemostasis technique used, it must be achieved quickly after removal of the inflamed pulp tissue. Its duration has no statistical effect on the success of cervical pulpotomy or on the intensity of postoperative pain [10,11]. However, it can be a diagnostic indicator for assessing the extent of inflammation and pulp healing capacity [12]. Some studies have suggested variable hemostasis duration ranging from 5 to 10 min [12], 2 to 25 min [15], or 1 to 10 min [17]. Thus, pulpotomy on mature permanent teeth with irreversible pulpitis has reshaped the fundamental concepts underlying the diagnosis and management of irreversible pulpitis [17].

Potential for healing: Some systematic reviews have shown that teeth affected by irreversible pulpitis have significant potential for healing because the inflammation is essentially limited to the coronal part of the pulp. The rest of the pulp may be intact, with the presence of Dental Pulp Stem Cells - Inflammatory Pulp "DPSC-IP", which have significant tissue regeneration potential. It also appears that pulpotomy modulates the pulp's immune response and reduces pro-inflammatory cytokines [9,16].

Periapical involvement: Discussed in four reviews [9,15,13,19], a radiographic lesion is generally associated with a lower success rate and postoperative pain. However, other reviews [9,15] indicate that the pulp may remain vital despite periapical inflammation, making diagnosis crucial.

Coronal restoration: Some reviews have indicated that coronal restoration is an important criterion in assessing the success of pulpotomy. Any lack of seal in this final coronal restoration is a major cause of pulpotomy failure. They found that prosthetic crowns and amalgam fillings are better than composite resin fillings [9,10].

Patient age: This factor does not appear to have any effect on the success of pulpotomy [12,15].

Summary: To increase the chances of successful pulpotomy on mature permanent teeth with irreversible pulpitis, strict adherence to the operating protocol, achievement of hemostasis, and, above all, a tight coronal filling are required.

Strengths and Limitations

The umbrella review has several strengths, including a transparent methodology, well-defined eligibility criteria, a rich corpus covering the short and medium term outcomes, and extensive comparative data on pulp-capping materials and their impact on treatment success. However, the review has several limitations, including low AMSTAR 2 quality, the lack of studies with a long follow-up period (over five years), and substantial heterogeneity among the included studies regarding methodology, population age, materials used, and criteria for assessing treatment success (clinical, radiological, or histological).

Impact of the Umbrella Review on Pediatric Clinical Practice

The first permanent molar is a tooth that is particularly susceptible to decay and can develop into irreversible pulpitis, which is a common dental problem in children. Although the majority of the included reviews focused on mixed populations, only three [9,12,15] explicitly included pediatric cohorts. They suggest that pulpotomy may be a viable therapeutic option for mature permanent teeth, provided there is an accurate diagnosis and adherence to the operative protocol. However, the data remain limited, which requires cautious interpretation and highlights the need for studies specifically focused on children.

Implications for Research

Based on this umbrella review, future research should focus on multicenter, methodologically rigorous randomized clinical trials with standardized diagnostic criteria and long-term follow-up exceeding five years, particularly in adolescents with mature permanent teeth. Our findings are in line with the recommendations of the American Association of Endodontists (AAE, 2021) [23] and the European Society of Endodontology (ESE, 2019) [24], which recognize pulpotomy as a viable treatment option under well-defined clinical conditions and support a shift toward more conservative endodontic therapies.

## Conclusions

This umbrella review highlights that pulpotomy is an effective treatment option for mature permanent teeth diagnosed with irreversible pulpitis, showing favorable clinical and radiographic outcomes comparable to conventional root canal treatment. Moreover, the choice of pulpotomy material may influence these outcomes, with calcium silicate-based hydraulic cements, such as MTA and Biodentine, providing superior and more consistent results. The findings support the growing role of vital pulp therapy in contemporary endodontic practice. Further high-quality randomized clinical trials with long-term follow-up are needed to strengthen the evidence base and guide clinical decision -making.
